# Amyloidosis: a case series and review of the literature

**DOI:** 10.1186/s13256-023-03886-1

**Published:** 2023-04-21

**Authors:** Justin B. Senecal, Romel Abou-Akl, Pat Allevato, Ian Mazzetti, Caroline Hamm, Richa Parikh, Indryas Woldie

**Affiliations:** 1 Schulich School of Medicine and Dentistry, London, ON Canada; 2 Windsor Regional Hospital, Windsor, ON Canada; 3 Karmanos Cancer Center, Detroit, MI USA

**Keywords:** Amyloidosis, Light chain, Monoclonal gammopathy

## Abstract

**Background:**

Systemic amyloidosis is group of disorders characterized by the accumulation of insoluble proteins in tissues. The most common form of systemic amyloidosis is light chain amyloidosis, which results from the accumulation of misfolded immunoglobulins. The disease is progressive, with treatment targeted at the underlying plasma cell dyscrasia. Since essentially any organ system can be affected, the presentation is variable and delays in diagnosis are common. Given this diagnostic difficulty, we discuss four different manifestations of light chain amyloidosis.

**Case presentations:**

In this case series, we discuss four cases of light chain amyloidosis. These include cardiac, hepatic, and gastrointestinal as well as autonomic and peripheral nerve involvement with amyloidosis. The patients in our series are of Caucasian background and include a  69-year-old female, a 29-year-old female, a 68-year-old male, and a 70-year-old male, respectively. The case discussions highlight variability in presentation and diagnostic challenges.

**Conclusions:**

Amyloidosis is a rare but serious disease that is often complicated by long delays in diagnosis. Morbidity and mortality can sometimes be limited if diagnosed earlier. We hope our real life cases will contribute to understanding and to early suspicion that can lead to early diagnosis and management.

## Background

Amyloidosis is a large group of diseases characterized by the accumulation of insoluble, misfolded proteins in tissues [1, 2]. These proteins can deposit locally, as in Alzheimer’s disease, or systemically. The clinical manifestations of the disease are caused by toxic effects of aggregated proteins deposited in the tissues [1]. The most common form of systemic amyloidosis is light chain amyloidosis (AL amyloidosis), which is characterized by accumulation of aggregated immunoglobulin light chain in tissues [1, 2]. This aberrant protein production most commonly results from a monoclonal plasma cell dyscrasia and rarely from other B-cell neoplasms. It is relatively rare, with an annual incidence of 10–14 cases/million [3, 4]. The median age at diagnosis is in the seventh decade of life and essentially any organ can be affected [3, 5–8]. The heart, peripheral nerves, kidneys, liver, and gastrointestinal tract are the most frequently involved, but mortality is often predicated on cardiac, renal, or autonomic nervous system dysfunction [2]. The natural history of the disease is almost always progressive, but at times can be reversible as aggregated protein is turned over [1, 2]. Treatment for all AL systemic amyloidosis is targeted at preventing further amyloidogenic protein production by treating the underlying plasma cell dyscrasia with chemotherapy [1, 2, 9].

The variation in presentation and rarity of AL amyloidosis makes it a challenging diagnosis to make; so much so that 37% of patients are not diagnosed until a year after presentation [10]. Early diagnosis is important, as advanced disease at the time of diagnosis likely contributes to 20% mortality in the first 6 months [11]. Given how often this disease is overlooked in clinical practice, it will be helpful to discuss presentation, diagnostic challenge, and management of real-life cases.

Verbal consent was obtained from two surviving patients and surviving spouse of another patient who died of amyloidosis-related complications. It was not possible to get consent for one patient who is currently deceased and whose immediate family member could not be traced.

## Case 1: Cardiac amyloid

### Clinical presentation

A 69-year-old Caucasian female with a past medical history significant for hypertension, gastroesophageal reflux disease, obstructive sleep apnea, and carpal tunnel syndrome was referred with new onset atrial fibrillation and congestive heart failure (CHF). She presented with fatigue, dyspnea on minimal exertion, and had recently been discharged from the hospital after an admission for acute on chronic CHF (NYHA III). Prior workup for coronary artery disease, including angiography, was unremarkable. She had been experiencing significant peripheral edema despite high doses of intravenous loop diuretics. There was no history of diabetes, chronic diarrhea, bleeding, or bruising. She had a 3-pack-year smoking history and admitted to drinking alcohol socially. Family history was notable for premature coronary artery disease (CAD) in both parents, cardiac arrest in a brother at age of 55, and another brother with a history of pulmonary embolism in the context of antiphospholipid antibody syndrome.

On examination, she was afebrile, hemodynamically stable, and had a body mass index (BMI) of 35. Head and neck exam was significant for a moderate macroglossia. No periorbital edema or lymphadenopathy were noted. Cardiac examination revealed irregular heart sounds and a mid systolic murmur. Auscultation of the chest revealed decreased air entry over the bases bilaterally. There was no hepatosplenomegaly. Bilateral pretibial edema was noted. Pertinent laboratory findings are summarized in Table 1.

**Table 1:** Pertinent laboratory findings

Pertinent investigations	Value (normal range)
WBC	15.7 × 10^9^/L (3.5–10.5)
ANC	10.8 × 10^9^/L (2.0–7.5)
Hemoglobin	123 g/L (115–155)
Platelets	343 × 10^3^/L (150–450)
Creatinine	107 mmol/L (< 75)
Ionized calcium	1.09 mmol/L (1.12–1.3)
Albumin	31.8 g/L (34–50)
Bilirubin	20 μmol/L (3–17)
LDH	340 IU/L (< 234)
Troponin	0.02 ng/L (< 0.1)
NT-proBNP	980 ng/L (< 100)
24-hour urine protein	0.22 g (< 0.2)
24-hour urine albumin	0.09 g (< 0.03)
*Gammopathy workup:*	
IgG	7.83 g/L (7–15)
IgM	0.72 g/L (0.6–3)
IgA	1.79 g/L (0.6–4)
Serum protein electrophoresis	No quantifiable monoclonal protein
Serum immunofixation	Discrete band of IgD lambda
Serum free light chains	Lambda: 186.4 mg/L (5.7–26.3) Kappa: 7.54 mg/L (3.3–19.4) Lambda/kappa: 24.6

*WBC* white blood cells, *ANC* absolute neutrophil count

### Imaging

Computed tomography (CT) chest with pulmonary embolism (PE) protocol was negative, but showed small-to-moderate bilateral pleural effusions. Abdominal ultrasound showed no hepatosplenomegaly. X-ray skeletal survey did not show any lytic lesions. A transthoracic echocardiogram showed mild concentric hypertrophy of the left ventricle (LV) with normal systolic function (ejection fraction of 65–70%), grade 1 diastolic dysfunction with impaired relaxation pattern, mild pulmonary hypertension [right ventricular systolic pressure (RVSP), 44 mmHg], moderate aortic sclerosis, mild bilateral atrial enlargement, interventricular septal enlargement at 12 mm (6–9 mm), and dilated inferior vena cava (IVC) with diminished inspiratory collapse consistent with increased right atrial pressure (RAP) of 15–20 mmHg (2–6 mmHg). Features suggestive of constrictive pathology were noted. Further workup with cardiac magnetic resonance imaging (MRI) showed mild concentric left ventricular (LV) hypertrophy. However, classic feature of cardiac amyloidosis (diffuse sub endocardial delayed enhancement) was not seen.

### Pathology

Abdominal fat pad biopsy showed benign fatty tissue with negative staining for Congo Red. Bone marrow aspiration and biopsy showed a slightly hypercellular marrow with 10% monoclonal (lambda-restricted) plasma cells. Further pathology revealed rare blood vessels within the periosteum with amorphous deposit positive for Congo Red stain, suggestive of amyloidosis. Proteomic analysis by mass spectrometry of Congo Red positive material indicated amyloid deposition consistent with light chain immunoglobulin type (AL) amyloidosis. Although cardiac MRI did not show classic sign of cardiac amyloidosis, right ventricular biopsy revealed features consistent with infiltrative cardiomyopathy with Congo Red staining positive for amyloid deposits, which was confirmed to be AL amyloidosis by mass spectrometry.

### Management and clinical course

Patient was diagnosed with systemic amyloidosis with bone marrow and cardiac involvement, stage III on the basis of elevated NT-proBNP [980 ng/L (< 100)] and troponin [0.02 ng/L (< 0.1)], with an associated median survival of 4.1 months [9]. Patient was enrolled in a phase III clinical trail with cyclophosphamide, bortezomib, and dexamethasone with or without daratumumab. Unfortunately, her disease course was complicated by acute on chronic renal impairment requiring hemodialysis 2 weeks into her treatment, and 2 weeks afterwards, the patient and her family decided to make her comfortable and she was transitioned to hospice.

### Discussion

Immunoglobulin D (IgD) monoclonal gammopathy is rare, affecting only 0.5% of patients with monoclonal gammopathy [12]. Interestingly, the presence of monoclonal IgD is almost always associated with multiple myeloma (MM), AL amyloidosis, or plasma cell leukemia; with only case reports of IgD monoclonal gammopathy of undetermined significance (MGUS) present in the literature [12]. Patients with IgD MM tend to present younger, have more severe disease, and are more likely to have extra medullary disease and poorer prognosis [13, 14]. Though not well studied, IgD AL amyloidosis is estimated to make up 1.3% of cases of AL amyloidosis and does not seem to have a survival disadvantage as in IgD MM [15]. However, patients with IgD MM are more likely to have concurrent AL amyloidosis (19–44%) when compared with non-IgD MM [16–18].

Cardiac amyloidosis is often the best predictor of mortality and morbidity [19] in systemic amyloidosis compared with other organ involvement [6]. It is an infiltrative disease that can be caused by many subtypes of systemic amyloidosis, with greater than 98% being caused by transthyretin (ATTR) or AL amyloidosis [20]. The former is reported to involve the heart in almost all cases, depending on the mutation, while the latter affects the heart 50–70% of the time [3, 20]. Cardiac AL amyloidosis can usually be diagnosed when there is evidence of monoclonal gammopathy, amyloidosis in another organs (usually via fat pad biopsy) and classic imaging evidence of cardiac involvement, particularly MRI. However, as in this case, ventricular biopsy is sometimes required for diagnosis, especially when classic MRI changes are not evident [3, 20]. Cardiac AL amyloidosis has a median survival of 24 months after diagnosis, compared to 31–69 months in ATTR amyloidosis [20]. The presence of heart failure at the time of diagnosis, as in this case, is associated with a poor median survival of 6 months [20]. Treatment is the same as with other organ involvement and is targeted at the underlying plasma cell dyscrasia [3, 20].

This patient presented with heart failure with preserved ejection fraction (HFpEF) and atrial fibrillation; two hallmarks of amyloid cardiac involvement [3, 9]. A past medical history of bilateral carpal tunnel and macroglossia on physical examination are also specific signs for systemic amyloidosis [3, 9]. Traditional echocardiography has a low sensitivity of 25–35% [21], and is particularly challenging in patients with hypertension, which can also cause concentric hypertrophy and HFpEF [21]. This patient had a negative cardiac MRI, despite its high sensitivity of 85–90% [7]. This highlights the importance of pursuing a tissue diagnosis when clinical suspicion is high. Interestingly, this patient also had a negative fat pad biopsy, which is positive in 78–100% of patients with cardiac AL amyloidosis [22].

Finally, family history of cardiac arrest in her brother at the age of 55 would have been a good reason to look for cardiac transthyretin (ATTR) amyloidosis, which is a familial genetic disorder. However, this patient had mass-spectrometry-confirmed cardiac AL amyloidosis, making further workup for other types of cardiac amyloid unnecessary.

### Learning points

1. HFpEF, new onset atrial fibrillation, and other conduction abnormalities are characteristic of cardiac amyloidosis.
2. Echocardiographic findings of amyloidosis can overlap with those found in hypertensive cardiomyopathy, and the diagnosis of amyloidosis can be missed without a high index of suspicion.
3. Cardiac MRI has a high sensitivity to diagnose AL amyloidosis, but a tissue biopsy should be pursued if clinical suspicion is high despite negative MRI findings.
4. IgD gammopathy is almost always associated with symptomatic disease and is rarely seen in asymptomatic patients with MGUS.

## Case 2: Hepatic amyloid

### Clinical presentation

A 29-year-old Caucasian female with a past medical history of hypertension, interstitial nephritis, and thin basement membrane disease leading to end stage renal disease (ESRD) requiring hemodialysis, presented with a 6-month history of progressive weight loss of about 20 kg over 6 months, fatigue, chronic diarrhea, and vomiting.

On examination, she was afebrile and hemodynamically stable. She was alert and oriented with an unremarkable neurological examination. There was no icterus or lymphadenopathy but mild pallor. Cardiac and respiratory exams were unremarkable. Labs are reviewed below (Table 2).

**Table 2:** Pertinent laboratory investigations at presentation

Pertinent investigations	Value (normal range)
WBC	9.5 × 10^9^/L (3.5–10.5)
ANC	6.7 × 10^9^/L (2.0–7.5)
Hemoglobin	97 g/L (115–155)
Reticulocyte	3.85 × 10^6^/L (0.5–2)
Platelets	385 × 10^3^/L (150–450)
Haptoglobin	0.97 g/L (0.5–2.2)
Ionized calcium	1.36 mmol/L (1.15–1.29)
PTH Creatinine ALT Alkaline phosphatase bilirubin Protein albumin NT-proBNP troponin INR aPTT	< 0.3 pg/mL (1.2–8.4) 418 umol/L (49–90) 14 u/L (11–61) 155 u/L (< 126) 18 umol/L (0–18) 66 g/L (60–80) 34 g/L (34–50) 2789 ng/L (< 100) 0.04 ng/L (< 0.1) 1.1 35 seconds (22–30)
LDH	219 IU/L (< 234)
Labs	
IgG	4.61 g/L (7–15)
IgM	0.65 g/L (0.6–3)
IgA	0.57 g/L (0.6–4)
Beta-2 macroglobulin	281 mg/L (6.78–19.8)
Serum protein electrophoresis	No monoclonal protein
Serum immunofixation	No monoclonal protein
Serum free light chains	Lambda: 1515 mg/L (5.7–26.3) Kappa: 96 mg/L (3.3–19.4) Lambda/kappa: 15.7

*WBC* white blood cells, *ANC* absolute neutrophil count, *LDH* lactate dehydrogenase, *PTH* parathyroid hormone

Laboratory findings were significant for a normocytic anemia, hypercalcemia, an elevated beta-2 microglobulin, and an elevated lambda/kappa ratio of 15.7 (Table 2). Liver enzymes were unremarkable and serology for HIV, hepatitis B, and hepatitis C were negative. Bone marrow aspiration and biopsy showed a normocellular marrow with 7–8% monoclonal plasma cells, lambda light chain restricted. Fluorescence *in situ* hybridization (FISH) revealed loss of TP53. Congo Red stain was negative for amyloidosis.

**Fig. 1:**
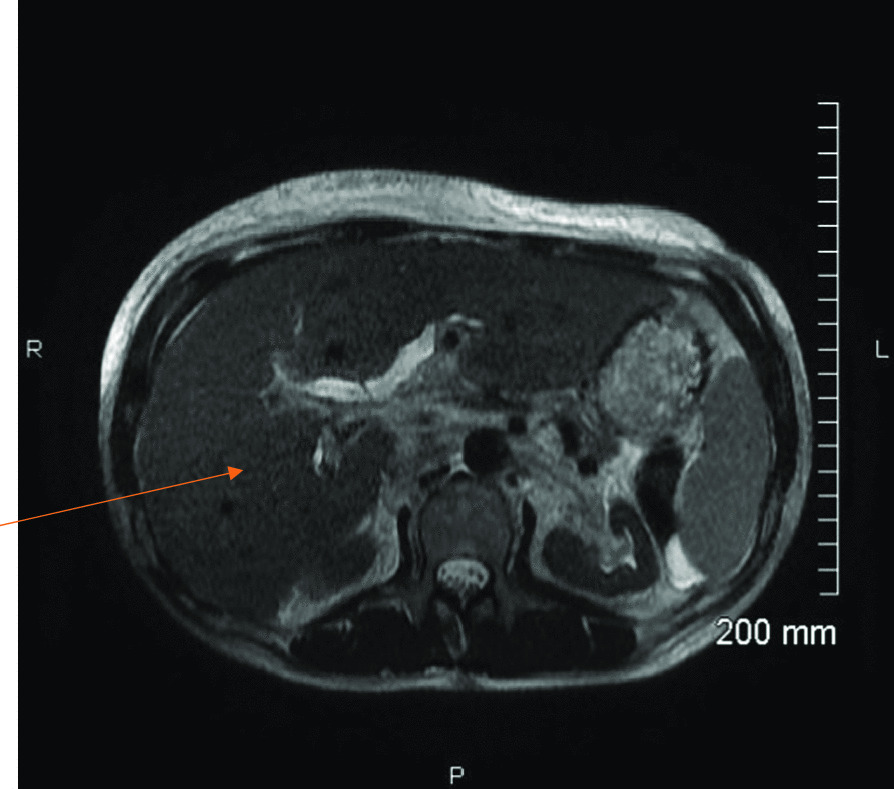
Magnetic resonance imaging liver showing diffuse hepatomegaly with no focal lesions (arrow)

With no clear diagnosis, a search for occult malignancy was initiated. CT neck, chest, abdomen, and pelvis demonstrated hepatomegaly with heterogeneous echotexture. Magnetic resonance cholangio pancreatography (MRCP) showed moderate intrahepatic duct dilatation and an enlarged liver measuring 25.5 cm in greatest dimension. Ultrasound (US)-guided liver biopsy revealed benign liver tissue with congested and dilated sinusoids with rare portal tracts showing amorphous deposits in the wall of the arteries (suggestive of amyloidosis) with focal Congo Red staining. Proteomic analysis by mass spectrometry of Congo Red positive arteries showed amyloid lambda light chain type. Positron emission tomography (PET)/CT showed massive hepatosplenomegaly without hypermetabolism, cardiomegaly, and diffuse biventricular hypermetabolism (Fig. 1).

Electrocardiogram (EKG) showed low-voltage QRS complexes. Transthoracic echocardiogram showed concentric left ventricular hypertrophy (LVH) with normal LV systolic function. It also showed speckled ground glass appearance of the myocardium consistent with amyloidosis. NT-proBNP was elevated at 2789 ng/L (< 100) but troponin-I was normal at 0.04 ng/L (< 0.1).

### Management and clinical course

Patient was diagnosed with systemic AL amyloidosis with liver (biopsy confirmed) and possibly cardiac involvement. She had complicated hospital course with cardiac arrest secondary to massive bleeding following liver biopsy and prolonged intensive care unit (ICU) stay. After recovery, she was treated with Cyclophosphamide, bortezomib, and dexamethasone. She had excellent biochemical response, with a 98% reduction in her involved free light chains after three cycles of chemotherapy. She also had resolution of hepatomegaly.

She was continued on dexamethasone and cyclophosphamide without bortezomib (due to peripheral neuropathy) with a plan to proceed with autologous stem cell transplantation at the time of relapse. She received a deceased donor renal transplant 2 years after initiation of treatment for amyloidosis with well functioning allograft to date. She is currently on ixazomib maintenance and in complete biochemical remission. It is now 4 years since her initial diagnosis. She is doing well and has resumed her full-time job.

### Discussion

This patient encountered diagnostic challenge (and delay) commonly seen in patients with systemic amyloidosis. Constitutional symptoms predominated her presentation, with hepatomegaly being the only localizing abnormality. Hepatic involvement is quite common among those with AL amyloidosis, with up to 90% of autopsies showing hepatic amyloid [23, 24]. Despite this prevalence, it is usually of minimal clinical significance. As in this case, constitutional symptoms such as anorexia, weight loss, and fatigue are common, but they reflect worsening systemic illness rather than liver dysfunction [25]. Stigmata of liver disease, including portal hypertension, are rare and are usually secondary to heart failure rather than hepatic amyloid [25].

Hepatomegaly and mildly elevated ALP (> 500) are the most common findings in patients with hepatic amyloid [26, 27]. Jaundice is rare but a poor prognostic sign [28]. Both morbidity and mortality in these patients is often due to disease elsewhere [25].

This patient suffered a major bleed during the hepatic biopsy. Some studies have reported an increased bleeding risk in patients with hepatic amyloid, but others have not [25]. Caution and close monitoring post-liver-biopsy is advised.

### Learning points

1. Hepatic amyloid is usually asymptomatic, with findings of hepatomegaly and elevated ALP.
2. Liver biopsy in patients with hepatic amyloid could be complicated with massive bleeding.
3. Relentless workup that led to proper diagnosis and management in this patient resulted in dramatic improvement.

## Case 3: Gastrointestinal (GI) amyloid

### Clinical presentation

A 68-year-old Caucasian male with a past medical history significant for peptic ulcer disease (PUD) was referred for workup and management of hypercalcemia. History was positive for significant fatigue. On physical examination, he was afebrile and hemodynamically stable. Chest was clear to auscultation with normal S1 and S2 on cardiac exam with no gallops or murmurs. He had no palpable hepatosplenomegaly or lymphadenopathy, and no focal neurologic deficits. Investigations are summarized in Table 3.

**Table 3:** Pertinent investigations at presentation including gammopathy workup

Pertinent investigations	Value (normal range)
WBC	5.5 × 10^9^/L (3.5–10.5)
ANC	3.8 × 10^9^/L (2.0–7.5)
Hemoglobin	112 g/L (115–155)
MCV	102 fL (80–100)
Platelets Creatinine	261 × 10^3^/L (150–450)
eGFR	46 (> 90)
Ionized calcium	1.68 mmol/L (1.12–1.3)
PTH	0.3 pg/mL (1.2–8.4)
Gammopathy workup	
IgG	29 g/L (7–15)
IgM	< 0.2 g/L (0.46–3.04)
IgA	< 0.31 g/L (0.92–4.53)
Beta-2 microglobulin	603.9 mg/L (67.8–198.3)
24-hour urine protein	5.36 g (< 0.2) with 99% gamma globulins
Urine protein electrophoresis	IgG kappa and free kappa
Serum protein electrophoresis Serum immunofixation	Monoclonal protein 20 g/L IgG kappa
Serum free light chains	Lambda: 3.8 mg/L (5.7–26.3) Kappa: 3620 mg/L (3.3–19.4) Kappa/lambda ratio: 952

*WBC* White Blood Cells, *ANC* Absolute Neutrophil Count, *MCV* Mean Corpuscular Volume, *PTH* Parathyroid Hormone

Skeletal survey did not show lytic lesions. Abdominal ultrasound revealed fatty liver changes, with no hepatosplenomegaly. Bone marrow aspiration and biopsy revealed 80% monoclonal plasma cells with standard risk cytogenetics.

### Management and clinical course

**Fig. 2:**
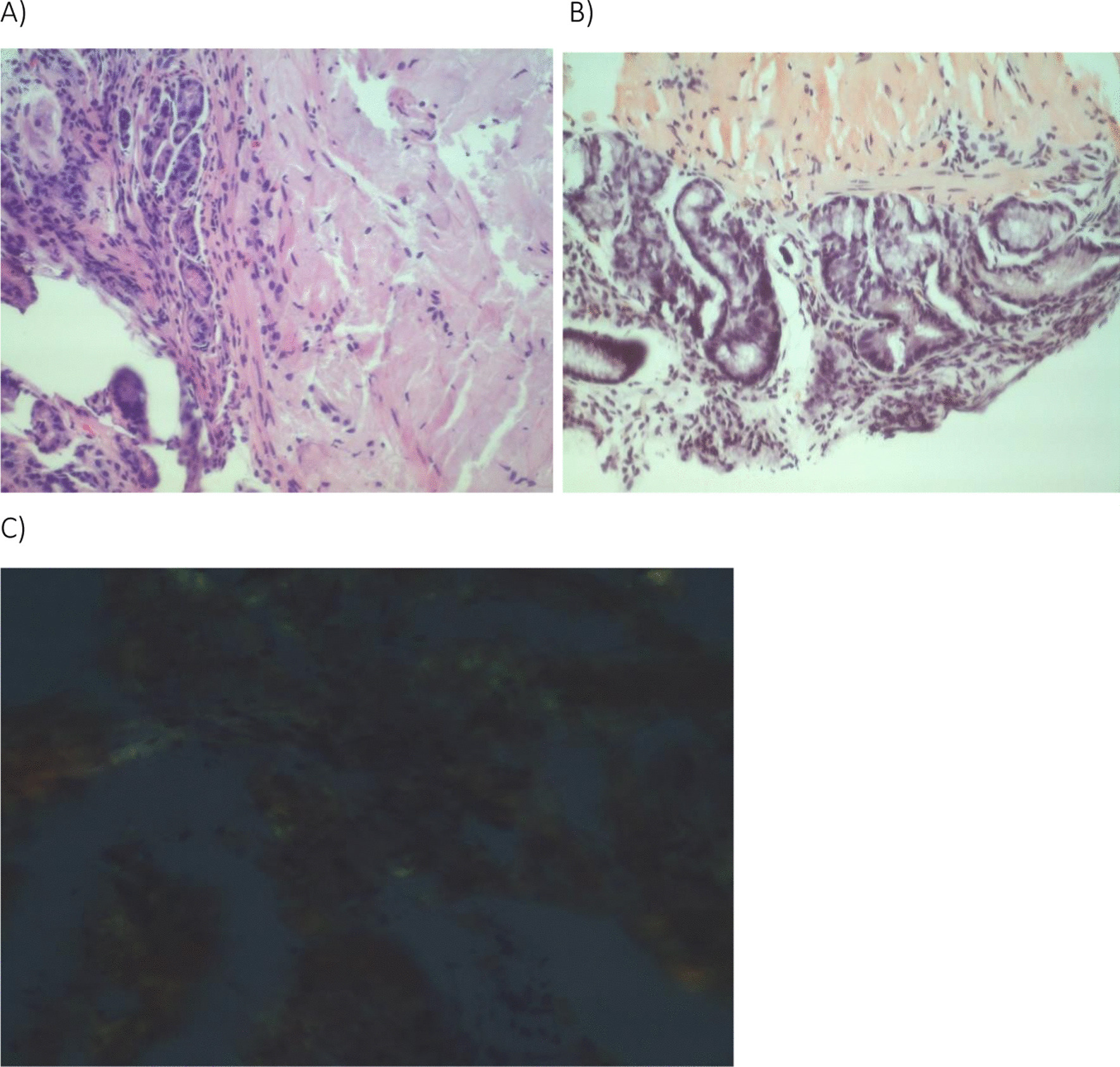
**A** H & E stain showing amorphous eosinophilic material within the gastric antral submucosa. **B** Congo Red stain of the amorphous material showing Red Orange color suggestive of amyloid. **C** Polarization shows “apple green” birefringence of amyloid

With the diagnosis of IgG kappa multiple myeloma, the patient was started on a cyclophosphamide-bortezomib-dexamethasone (CyBorD) chemotherapy regimen. After three doses, the patient developed severe nausea and vomiting requiring hospital admission. An abdominal X-ray and barium swallow were normal. He was thought to have steroid-induced gastritis. He was given a prescription for antiemetics and proton pump inhibitor (PPI) twice daily. The dose of dexamethasone was reduced to 10 mg weekly. Subsequently, he noted some improvement in his symptoms but later developed diarrhea, not relieved with antidiarrheal agents. He underwent esophagogastroduodenoscopy (EGD) and colonoscopy, which showed erosive gastritis, two small pyloric ulcers, and diverticular left colon with melanosis coli, respectively. Biopsy from gastric mucosa and duodenum showed interstitial amyloid deposits confirmed to be AL amyloidosis by mass spectrometry (Fig. 2).

A cardiac workup was pursued. ECG revealed low-voltage QRS complex. Transthoracic echocardiogram demonstrated intraventricular septum thickness of 1.75 cm. Cardiac MRI revealed normal global and regional left and right ventricular systolic function, with no evidence of abnormal myocardial delayed contrast enhancement. There was moderate circumferential pericardial effusion. Right ventricular biopsy showed postinflammatory dilated cardiomyopathy, with negative Congo Red staining.

The patient continued on the same regimen (CyBorD) and completed six cycles of chemotherapy. He achieved a very good partial response (VGPR) and proceeded with autologous stem cell transplantation using melphalan 200 mg/m^2^ conditioning and was then kept on lenalidomide maintenance. It is now 3.5 years since his initial diagnosis and he is alive and doing well.

### Discussion

Symptomatic GI amyloidosis has a 1% incidence in primary systemic amyloidosis [29]. GI amyloid can present in the secondary AA subtype or the primary AL subtype as in this case. Prognosis varies depending on the etiology of the disease, with the AL subtype, as well as those with hepatic involvement, showing worse outcomes [30]. Within the GI tract, symptoms of AL amyloidosis are often nonspecific and range from nausea, reflux, and diarrhea to more severe manifestations such as hemorrhage and obstruction [30].

This patient did not present with any GI symptoms initially, but experienced more severe nausea, vomiting, and diarrhea than one would expect from the chemotherapy. He did have a past medical history significant for peptic ulcers, which could have been a manifestation of amyloidosis as well [31].

### Learning points

1. GI symptoms (nausea, vomiting, diarrhea, and so on) from chemotherapy that are more than expected can point to a diagnosis of AL amyloidosis in patients with known MM or MGUS.
2. Most patients with GI amyloidosis are asymptomatic. However, if symptomatic, they typically present with nonspecific symptoms such as nausea, vomiting, reflux, and diarrhea.
3. Amyloid deposits may cause recurrent gastric ulcers, and thus, such history should arouse suspicion for GI amyloidosis in patients with known MM or MGUS.

## Case 4: Progressive peripheral neuropathy, autonomic neuropathy, and anorexia in AL amyloidosis

### Initial presentation

76-year-old Caucasian male with known IgG lambda monoclonal gammopathy and peptic ulcer disease presented with a 3-year history of paresthesia initially involving bilateral lower extremities with subsequent extension to bilateral upper extremities. His symptoms progressively worsened and he developed significant muscle wasting and bilateral foot drop. He also developed orthostatic hypotension that prevented him from ambulating independently. His main compliant was altered taste sensation leading to anorexia and around 30-pound weight loss over a period of 2 years.

He was evaluated by several specialties over a period of 3 years and had extensive workup without definitive diagnosis. Autoimmune, paraneoplastic, and extensive neurologic workup were all negative. At one point, the possibility of distal sensory motor neuropathy related to DNAIB2 sequence variant found on Charcot-Marie-Tooth panel screen was considered as a working diagnosis and he was being followed by neurology for a while. Eventually he was referred to hematology for possible amyloidosis.

Review of systems was negative for diarrhea, bleeding, bruising, orthopnea, paroxysmal nocturnal dyspnea (PND), or peripheral edema. He was a lifelong nonsmoker with an unremarkable family history.

On examination, he was afebrile. Orthostatic hypotension was noted. He appeared frail. Neurological exam revealed generalized muscle wasting involving the upper and lower extremities, bilateral foot drop, and decreased sensation to light touch in both upper and lower extremities in a stock and glove pattern. There was no palpable lymphadenopathy. Cardiorespiratory exam was unremarkable. His abdomen was soft, non-tender, and non-distended with no palpable hepatosplenomegaly. Investigations are reviewed in Table 4.

**Table 4:** Pertinent investigations

Pertinent investigations	Value (normal range)
WBC	10 × 10^9^/L (3.5–10.5)
Hemoglobin	118 g/L (115–155)
Platelets	290 × 10^3^/L (150–450)
Ionized calcium	1.19 mmol/L (1.15–1.29)
24-hour urine protein troponin-I NT-proBNP	0.55 g/day (< 0.15) 0.54 ng/L (< 0.09) 357 ng/L (< 100)
*Gammopathy workup*	
IgG IgM IgA	15.6 g/L (7–16) 0.36 g/L (0.46–3) 2.01 g/L (0.82–4.5)
Beta-2 microglobulin	172 mg/L (67.8–198.3)
Serum protein electrophoresis	Monoclonal protein of 5 g/L
Serum immunofixation	IgG lambda
Serum free light chains	Lambda: 66.9 mg/L (5.7–26.3) Kappa: 22.4 mg/L (3.3–19.4) Lambda/kappa: 2.99

*WBC* white blood cells

Initial laboratory findings were significant for an IgG lambda monoclonal gammopathy. Bone marrow biopsy (which was negative 3 years prior) showed mild, lambda restricted, plasmacytosis with positive Congo Red stain, suggesting a diagnosis of amyloidosis. Echocardiogram showed normal LV systolic function with an EF of 65%, grade I diastolic dysfunction, and IVSD of 10 mm. Troponin-I was mildly elevated at 0.54 ng/L (< 0.09) and NT-proBNP was elevated at 357 ng/L (< 100).

Given weight loss, esophagogastroduodenoscopy was performed that showed reactive gastropathy. Gastric biopsies were positive for Congo Red with mass spectrometry confirming lambda type amyloid deposition.

### Management and clinical course

The diagnosis of systemic AL amyloidosis with confirmed GI and bone marrow as well as possible cardiac, autonomic, and peripheral nerve involvement was made, and he was started on chemotherapy using CyBordD regimen. Unfortunately, he was admitted to the ICU shortly after initiation of chemotherapy with severe sepsis, was on mechanical ventilation as well as multiple pressors, and finally succumbed to his disease.

### Discussion

Here we present a case of systemic AL amyloid that presented with predominant neurological findings and a diagnostic delay of 3 years. This diagnostic delay is not uncommon among those presenting with neuropathy, with diagnosis taking 29–48 months on average [32]. This delay is concerning as those with only neuropathy are often the most suitable for intensive treatment [33].

Polyneuropathy, organomegaly, endocrinopathy, monoclonal gammopathy, skin changes (POEMS) syndrome is a rare disorder that also falls under the umbrella of plasma-cell-related disorders. It can be difficult to differentiate from AL amyloidosis, with amyloid protein not excluding POEMs syndrome [34, 35]. Polyneuropathy in the context of an M protein is the defining characteristic of POEMS syndrome [34]. The features included in the acronym are important but are neither required nor the only manifestations of this disease [34]. The gammopathy in POEMS syndrome is almost always lambda restricted [36]. The polyneuropathy in both AL amyloid and POEMS is frequently bilateral, painful, and symmetrical with both sensory and motor findings [33, 34]. Autonomic involvement, as in this case, is more suggestive of amyloidosis than POEMS syndrome [33]. Characteristics that favor POEMS syndrome over AL amyloidosis include coexisting thrombocytosis/erythrocytosis, elevated VEGF, endocrinopathy, and bone marrow biopsy with megakaryocytic hyperplasia and clustering, or plasma cell rimming of lymphoid aggregates [34].

This patient presented with profound weight loss, which is the most common clinical manifestation of GI amyloidosis [3, 37]. It is unclear how much of this weight loss was due to anorexia from dysgeusia, which is a phenomenon of systemic amyloidosis that has been described with macroglossia [38]. In this patient, delayed diagnosis most likely resulted in adverse outcome, indicating the need to be vigilant and initiate workup for systemic amyloidosis.

### Learning points

1. Neuropathy is a common presentation in AL amyloidosis. Differential diagnoses includes POEMS syndrome and multiple myeloma.
2. Obtaining a tissue diagnosis is often the only way to differentiate between AL amyloidosis and other causes of neuropathy associated with plasma cell dyscrasias.
3. Delay in diagnosis of systemic amyloidosis leads to adverse outcomes that could have been potentially prevented.

## Conclusion

Although not common, amyloidosis is a serious disease that results in significant mortality and morbidity. The most challenging issue is significant delay in diagnosis, sometimes up to several years. Early suspicion and thorough investigation are critical to making a timely diagnosis and referral for treatment. We believe reporting real life cases such as ours will provide valuable insight for health professionals who may come across similar patients during their practice.

## Data Availability

All data generated or analyzed during this study are included in this published article.
